# Age at menarche and risk of major cardiovascular diseases: Evidence of birth cohort effects from a prospective study of 300,000 Chinese women

**DOI:** 10.1016/j.ijcard.2016.10.115

**Published:** 2017-01-15

**Authors:** Ling Yang, Liming Li, Iona Y. Millwood, Sanne A.E. Peters, Yiping Chen, Yu Guo, Zheng Bian, Xiaofang Chen, Lingli Chen, Shixian Feng, Silu Lv, Zhigang Pang, Mark Woodward, Zhengming Chen, on behalf of the China Kadoorie Biobank study collaborative group (members listed in the acknowledgement section)

**Affiliations:** a Clinical Trial Service Unit and Epidemiological Studies Unit (CTSU), University of Oxford, Oxford, UK; b Chinese Academy of Medical Sciences, Dong Cheng District, Beijing, China; c Department of Public Health, Beijing University, Beijing, China; d The George Institute for Global Health, University of Oxford, Oxford, UK; e Pengzhou CDC NCDs Prevention and Control Department, Penzhou, Sichuan, China; f Tongxiang CDC NCDs Prevention and Control Department, Tongxiang, Zhejiang, China; g Henan CDC NCDs Prevention and Control Department, Zhengzhou, Henan, China; h Licang CDC, Qingdao, Shandong, China; i Heilongjiang CDC NCDs Prevention and Control Department, Harbin, Heilongjiang, China; j The George Institute for Global Health, University of Sydney, Australia; k Department of Epidemiology, Johns Hopkins University, Baltimore, MD, USA

**Keywords:** Age at menarche, Cardiovascular disease, Prospective studies, Birth cohort, Chinese

## Abstract

**Background:**

Previous studies of mostly Western women have reported inconsistent findings on the association between age at menarche and risk of cardiovascular disease (CVD). Little is known about the association in China where there has been a large intergenerational decrease in women's mean age at menarche.

**Methods:**

The China Kadoorie Biobank recruited 302,632 women aged 30–79 (mean 50.5) years in 2004–8 from 10 diverse regional sites across China. During 7 years follow-up, 14,111 incident cases of stroke, 14,093 of coronary heart disease (CHD), and 3200 CVD deaths were reported among 281,491 women who had no prior history of CVD at baseline. Cox regression yielded adjusted hazard ratios (HRs) relating age at menarche to CVD risks.

**Results:**

The mean (SD) age of menarche was 15.4 (1.9) years, decreasing from 16.2 (2.0) among women born before 1940 to 14.7 (1.6) for those born during the 1960s–1970s. The patterns of association between age at menarche and CVD risk appeared to differ between different birth cohorts, with null associations in older generations but U-shaped or weak positive associations in younger women, especially those born after the 1960s. After minimizing the potential confounding effects from major CVD risk factors, both early and late menarche, compared with menarche at age 13 years, were associated with increased risk of CVD morbidity and mortality, which was more pronounced in younger generations.

**Conclusion:**

Among Chinese women the associations between age at menarche and risk of CVD differed by birth cohort, suggesting other factors may underpin the association.

## 1 Introduction

Cardiovascular disease (CVD) is a leading cause of mortality and morbidity throughout the world, including China [1]. The incidence of CVD is low in women of reproductive age but rises rapidly after menopause, leading to the ‘oestrogen hypothesis’ which suggests that endogenous oestrogen protects women of reproductive age from CVD. [2], [3] Menarche, defined as the first menstrual period signifies the onset of reproductive capacity, and its timing may have important health implications, either directly or indirectly.

There is evidence that early menarche is associated with certain CVD risk factors, such as increased body mass index (BMI) [4], [5]. Several studies of mostly Western populations have examined the association of early menarche with CVD, but the findings have been inconsistent, with some showing no associations, while others reported a U-shaped association, especially in more recent studies in which a high proportion of women had age at menarche before or during the early teenage years [5], [6], [7], [8]. Evidence from Asian populations, particularly from Chinese women, where the mean age at menarche tended to be later compared with their counterparts in the West, is limited [9], [10], [11]. A few small studies in Asia, where most women were born during the first half of 20th century and had experienced their first menstrual period during their late teenage years (mean age at menarche over 16 years) suggested that the patterns of association may differ from that in Western populations, for reasons that are not properly understood [10], [11]. In China there has been a rapid change in women's reproductive patterns in recent decades, [12] which may well have long-term health consequences. We examined the association between age at menarche and risk of major CVDs and investigated intergenerational differences in 300,000 women from the China Kadoorie Biobank (CKB) prospective study who were born during the 1920s–1970s.

## 2 Methods

### 2.1 Study population

Detailed information about the CKB study design and procedures has been reported previously [13]. Briefly, the baseline survey took place during 2004–8 in 10 geographically defined areas across China Overall, 512, 891 individuals (302,632 women) aged 30–79 years were enrolled. All participants provided written consent for follow-up. International, national, and local ethics approval was obtained.

### 2.2 Data collection

At study assessment clinics, trained health workers administered laptop-based questionnaires on socio-demographic status, dietary and other lifestyle habits, personal and family history. A range of physical measurements, including blood pressure and anthropometry, were undertaken for each participant along with collection of a blood sample for long-term storage. Every 4–5 years a 5–6% random sample of surviving participants was resurveyed.

### 2.3 Women's reproductive history

Women's reproductive history included age at menarche, parity, age at birth and breastfeeding duration for each live birth, menopause status and age at menopause for post-menopausal women, and history of oral contraceptive (OC) use, hysterectomy, ovarian or breast surgery.

### 2.4 Follow-up for mortality and morbidity

Since recruitment, participants have been followed-up for morbidity and mortality through linkage with regional disease and death registers, and for all hospitalized events through electronic data linkage with the nationwide health insurance system. Causes of death are sought chiefly from official death certificates, supplemented, if necessary, by reviewing medical records. To minimize losses to follow-up, active follow-up is also performed annually [13]. In the present study the main outcome measures were CVD deaths (ICD-10: I00-I99), fatal or non-fatal CHD (I20-I25) and stroke (I60-I69). Subtypes of stroke, i.e. ischemic (I63) or hemorrhagic stroke (I61) were also assessed.

### 2.5 Statistical analyses

Among 302,632 women recruited at baseline, we excluded 2012 women with inconsistent or extreme data for any reproductive factor, and 19,129 women who had a prior history of CVD, cancer or oophorectomy. After these exclusions, 281,491 women remained in the main analyses.

Cox proportional hazards models were used to estimate hazard ratios (HRs) for CVD in relation to age at menarche, stratified by region, age at risk, and education (Model 1). Further adjustments were made (Model 2) for conventional CVD risk factors, including systolic blood pressure, household income, smoking, alcohol drinking, BMI, physical activities (metabolic equivalent tasks [METs-hrs/day]) [14], self-reported or screen-detected diabetes, and leg length as a marker of pre-pubertal growth [15]. To minimize potential confounding by other reproductive factors [10], [16], [17], [18], [19], we additionally adjusted for menopause status, parity, age at first birth, breastfeeding duration and OC use (Model 3). We also conducted analyses among women who had never smoked or consumed alcohol, had no diabetes at baseline and never used OCs (Model 4). Given the striking temporal trends in reproductive factors we reported previously [12], further analyses were done by birth cohort to assess whether the associations between age at menarche and CVD risk varied significantly by birth cohort. The 95% confidence interval (CI) for each log HR was estimated using the ‘floating absolute risk’ method [20]. To correct for regression dilution bias related to reporting error in age at menarche [21], [22], HRs in the groups determined at baseline were plotted against the usual menarche age, i.e. the mean value of menarche age in that group at the 2008 resurvey. Analyses were performed using SAS version 9.3 and R version 3.0.1.

## 3 Results

Among the 281,491 women included, the mean (SD) age was 50.5 (10.3) years, 43% were urban residents, few ever smoked (2%), drank alcohol regularly (2%) or used OCs (10%) but nearly all had given birth (98.7%) and breastfed their children (97.3%) (Table 1). Overall the mean age at menarche was 15.4 (1.9) years, with a large decrease across generations, from 16.2 (2.0) years among women born before 1950 to 15.6 (1.9) years in the 1950s and 14.7 (1.7) years in those born after 1959.

Compared to women with a later onset of menarche, those who had early menarche (i.e. ≤ 12 years) were, on average, younger, more likely to be urban residents, better educated, to have higher income, higher mean BMI and blood pressure, shorter leg length, more likely to be physically inactive and to have self-reported diabetes. Women with earlier menarche also tended to have a later age at first birth, shorter duration of breastfeeding, earlier age at menopause and higher number of reproductive years than women with later menarche (Table 1).

During 2.0 million women-years of follow-up, 14,111 participants had stroke (including 11,173 ischemic, 2142 hemorrhagic) 14,093 had CHD (including 1177 acute myocardial infarction), and 3200 died from CVD.

### 3.1 Age at menarche and stroke

**Fig. 1:**
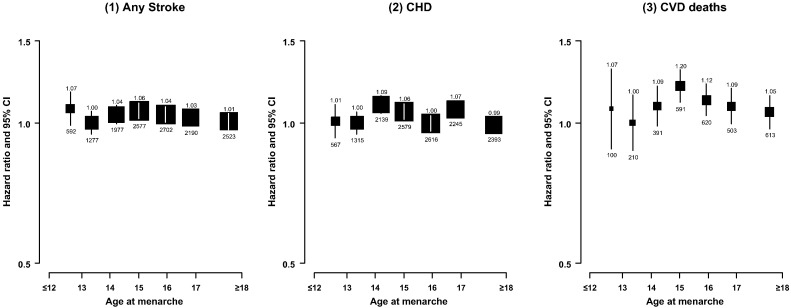
Adjusted hazard ratios for stroke, CHD and CVD deaths associated with age at menarche. Analyses were stratified by age and study area, and adjusted for education, measured blood pressure, household income, smoking, alcohol drinking, BMI, physical activities (MET), prior-diabetes status and leg length. The hazard ratios (HRs) are plotted on a floating absolute scale, and plotted against mean usual age at menarche level in each category. Squares represent the HR with area inversely proportional to the variance of the log HR. Vertical lines indicate the corresponding 95% confidence intervals (CIs). Numbers above CIs are of HRs and those below are the numbers of studied events.

Overall, age at menarche was not significantly associated with total stroke (Fig. 1) or stroke subtypes (web-Figure), after adjustment for lifestyle risk factors both overall and in subgroups of women defined by residential area, overweight status or education level (web-Tables 1, 2). Further adjustment for other reproductive factors did not alter the results substantially. However, the association appeared to vary considerably by birth cohort. Early menarche was associated with 16% lower risk of stroke among women born during the 1920s–1940s, but conversely with 18% increased risk among women born in the 1950, compared with menarche at age 13–14 years. No association of stroke with later ages at menarche was observed in women born before 1959. Among women who were born in the 1960s–1970s, however, there was a U-shaped association with HRs of 1.24 (1.05–1.47), 1.00 (0.92–1.09) 1.12 (1.03–1.21) and 1.21 (1.07–1.37), respectively for those with age of menarche at ≤ 12, 13–14, 15–16 and ≥ 17 years (*P*_*interaction*_ = 0.02) (Table 2). This birth cohort effect may contribute to the different associations observed in subgroups defined by menopause status. There was a slight U-shaped association, with a nadir at 13 years, among pre-menopausal women (median birth year of 1964), while a null association among post-menopausal women (median birth year of 1949) (*P*_*interaction*_ = 0.007). After excluding women with major CVD risk factors (Model 4), both early and later menarche were associated with increased risks of stroke, compared with women with menarche at age 13 years (web-Table 1). However, this U-shaped association was seen in younger generations but not in older women (data not shown).

### 3.2 Age at menarche and CHD

No association was observed between age at menarche and risk of CHD overall (Fig. 1) or in subgroups of women (web-Table 2). There was no clear association between age at menarche and CHD risk among women born before 1959, however among those born during the 1960s–1970s risk of CHD increased slightly with increasing age at menarche (Table 2). Both early and late menarche, compared with menarche at age 13–14 years, slightly increased risk of CHD among women with no major CVD risk factors and in subgroups defined by region, education, BMI and menopausal status (web-Tables 1,2).

### 3.3 Age at menarche and CVD deaths

As for stroke and CHD risk, overall there was a null association between age at menarche and risk of CVD mortality (Fig. 1). In further analyses by birth cohort, there was no association between age at menarche and risk of CVD death in those born in the 1920s–1940s, but a weak positive association was present in younger women born in the 1950s–1970s (Table 2). Either early or later menarche was associated with increased risks of CVD death in women with no major CVD risk factors, with adjusted HRs of 1.37 (1.03–1.81), 1.00 (0.81–1.23), 1.22 (1.05–1.41), 1.35 (1.21–1.51), 1.29 (1.16–1.44), 1.26 (1.11–1.42) and 1.24 (1.11–1.39) for those with menarche age at < 13, 13, 14, 15, 16, 17 and ≥ 18 respectively. This U-shaped association was seen in subgroups defined by birth cohort and other CVD risk factors, although the numbers of cases were small (data not shown).

## 4 Discussion

This is the first large prospective study of the association between age at menarche and the risk of CVD in China. Among about 300,000 women who were recruited from the general communities in 10 diverse regions of China, the mean age of menarche showed large intergenerational decreases. The patterns of association between age at menarche and CVD risks appeared to differ somewhat between different birth cohorts, with null associations in older generations but U-shaped or weak positive associations in younger women, especially those born after the 1960s. After minimizing potential confounding from major CVD risk factors, both early and late menarche were associated with increased risk of CVD morbidity and mortality, compared with menarche at age 13–14 years. Our findings suggest that among Chinese women the risk of CVD associated with pubertal timing may be influenced by factors such as socio-economic status, adiposity, and blood pressure.

Previous studies of mostly Western women born before the 1960s with mean age at menarche below 13 years, have reported inconsistent findings on the association between age at menarche and CVD risks [2], [5], [6], [7], [8], [9], [19], [23]. A recent report from the UK Million Women Study (MWS) of 1.2 million post-menopausal British women born between 1930–1950s with 73,378 CHD and 25,426 stroke cases, showed a clear U-shaped association for both diseases. In that study, women who had menarche ≤ 10 years or ≥ 17 years both had about 25% excess risk for CHD and 15% excess risk for stroke, compared with those with menarche age of 13 years. [7] A similar U-shaped association between age at menarche and prevalent CVD was also seen among 250,000 women in the UK-Biobank cohort [23]. However, neither of these studies investigated birth cohort effects. In our study, we find a similar U-shaped association for stroke among women born after the 1960s, who, compared with older generations, had more similar characteristics on both lifestyle and reproductive factors as in Western women.

Limited data from other studies in China also suggested different patterns of association of age at menarche with CVD by birth cohort. In the Shanghai Women's Health study of 31,955 post-menopausal women (most born before the 1940s), there was no association between age at menarche and total CVD and CHD mortality. [24] In contrast, the Shanghai female textile workers study of 267,400 women (60% born after 1950) found that early menarche (< 14 vs. > 14 years) was associated with 44% increased risk of CHD (1.44 [1.00–2.05]), but no excess stroke mortality [10]. Compared with women born in the 1920s–30s in China, younger generations tended to be much better educated, less likely to smoke and drink alcohol, more physically active and to have fewer children and shorter breastfeeding duration (web-Table 3). It is likely that the pattern of reproductive factors and their interplay with lifestyle factors may affect subsequent associations of reproductive factors with CVD risks. Indeed, when we excluded the confounding effects from some major CVD risk factors, U-shaped associations between age at menarche and CVD risk were present, which were more pronounced in younger generations. The rapid industrialisation of China with increasing wealth has resulted in increased prevalence of CVD and CVD risk factors, increasingly mirroring those seen in Western populations. Women who experienced early menarche are likely to have higher BMI, higher blood pressure, increased risk of insulin resistance, and metabolic syndrome [5]. If the increased risk of stroke with early menarche seen in our study, particularly in the younger women, was casual, the shift towards younger age at menarche over time would result in an increased burden of stroke in China over the next few decades; on the other hand, preventing the risk factors of early age at menarche, such as childhood obesity, could have public health benefits.

The mechanisms underlying variation in the timing of menarche are unclear. Genome-wide association studies on the timing of menarche have been conducted in European women, with over 100 genomic loci associated with age at menarche identified which also overlap somewhat with genes implicated in BMI and various diseases. [25], [26], [27] This suggests there may be shared causal pathways between age at menarche and adiposity influencing disease risks. However, limited genetic evidence has been reported from Asian women.

Our study has a number of strengths including large sample size, good quality and completeness of data collected and wide range of reproductive history information. Moreover, there was high repeatability of age at menarche (i.e. intra-class correlation coefficient of 0.84 between the baseline survey and the resurvey) and a high accuracy (~ 90%) of the reported diagnoses based on our ongoing CVD outcome adjudication. Several limitations need to be taken into account. Lack of information on childhood adiposity precluded assessment of the effects of adiposity at an earlier age, which has been closely associated with pubertal timing [5]. However, adiposity in later adulthood, which we were able to adjust for, may be more likely to indicate long-term exposure to being overweight, and thus may be more important when assessing the increased CVD risk associated with early menarche. Residual confounding from other unknown risk factors may exist due to the observational nature of the study.

The different associations between age at menarche and CVD risk among different populations and between birth cohorts within a population, as observed in our study, have implications for future research. Findings from one population may not be applicable to another that has a different distribution of age at menarche, thus, systematic reviews or meta-analyses on this topic should take into account these varying distributions in populations from different studies. Further investigation from both observational and genetic studies is needed on the underlying reasons for differences in the patterns of association between age at menarche and disease risk, and interplay with related generational, life-style or other reproductive factors.

## Members of the China Kadoorie Biobank collaborative group

International Steering Committee: Junshi Chen, Zhengming Chen (PI), Rory Collins, Liming Li (PI), Richard Peto.

International Co-ordinating Centre, Oxford: Daniel Avery, Derrick Bennett, Yumei Chang, Yiping Chen, Zhengming Chen, Robert Clarke, Huaidong Du, Xuejuan Fan, Simon Gilbert, Alex Hacker, Michael Holmes, Andri Iona, Christiana Kartsonaki; Rene Kerosi, Ling Kong, Om Kurmi, Garry Lancaster, Sarah Lewington, John McDonnell, Winnie Mei, Iona Millwood, Qunhua Nie, Jayakrishnan Radhakrishnan, Sajjad Rafiq, Paul Ryder, Sam Sansome, Dan Schmidt, Paul Sherliker, Rajani Sohoni, Iain Turnbull, Robin Walters, Jenny Wang, Lin Wang, Ling Yang, Xiaoming Yang.

National Co-ordinating Centre, Beijing: Zheng Bian, Ge Chen, Yu Guo, Bingyang Han, Can Hou, Jun. Lv, Pei Pei, Shuzhen Qu, Yunlong Tan, Canqing Yu, Huiyan Zhou.

### 10 Regional co-ordinating centres

Qingdao Qingdao CDC: Zengchang Pang, Ruqin Gao, Shaojie Wang, Yongmei Liu, Ranran Du, Yajing Zang, Liang Cheng, Xiaocao Tian, Hua Zhang. Licang CDC: Silu Lv, Junzheng Wang, Wei Hou.

Heilongjiang Provincial CDC: Jiyuan Yin, Ge Jiang, Shumei Liu, Zhigang Pang, Xue Zhou. Nangang CDC: Liqiu Yang, Hui He, Bo Yu, Yanjie Li, Huaiyi Mu, Qinai Xu, Meiling Dou, Jiaojiao Ren.

Hainan Provincial CDC: Jianwei Du, Shanqing Wang, Ximin Hu, Hongmei Wang, Jinyan Chen, Yan Fu, Zhenwang Fu, Xiaohuan Wang, Hua Dong. Meilan CDC: Min Weng, Xiangyang Zheng, Yijun Li, Huimei Li, Chenglong Li.

Jiangsu Provincial CDC: Ming Wu, Jinyi Zhou, Ran Tao, Jie Yang. Suzhou CDC: Jie Shen, Yihe Hu, Yan Lu, Yan Gao, Liangcai Ma, Renxian Zhou, Aiyu Tang, Shuo Zhang, Jianrong Jin.

Guangxi Provincial CDC: Zhenzhu Tang, Naying Chen, Ying Huang. Liuzhou CDC: Mingqiang Li, Jinhuai Meng, Rong Pan, Qilian Jiang, Jingxin Qing, Weiyuan Zhang, Yun Liu, Liuping Wei, Liyuan Zhou, Ningyu Chen, Jun. Yang, Hairong Guan.

Sichuan Provincial CDC: Xianping Wu, Ningmei Zhang, Xiaofang Chen, Xuefeng Tang. Pengzhou CDC: Guojin Luo, Jianguo Li, Xiaofang Chen, Jian Wang, Jiaqiu Liu, Qiang Sun.

Gansu Provincial CDC: Pengfei Ge, Xiaolan Ren, Caixia Dong. Maiji CDC: Hui Zhang, Enke Mao, Xiaoping Wang, Tao Wang.

Henan Provincial CDC: Guohua Liu, Baoyu Zhu, Gang Zhou, Shixian Feng, Liang Chang, Lei Fan. Huixian CDC: Yulian Gao, Tianyou He, Li Jiang, Huarong Sun, Pan He, Chen Hu, Qiannan Lv, Xukui Zhang.

Zhejiang Provincial CDC: Min Yu, Ruying Hu, Le Fang, Hao Wang. Tongxiang CDC: Yijian Qian, Chunmei Wang, Kaixue Xie, Lingli Chen, Yaxing Pan, Dongxia Pan.

Hunan Provincial CDC: Yuelong Huang, Biyun Chen, Donghui Jin, Huilin Liu, Zhongxi Fu, Qiaohua Xu. Liuyang CDC: Xin Xu, Youping Xiong, Weifang Jia, Xianzhi Li, Libo Zhang, Zhe Qiu.

## Funding/funders

The CKB baseline survey and the first re-survey were supported by the Kadoorie Charitable Foundation in Hong Kong. The long-term follow-up is supported by the UK Wellcome Trust [088158/Z/09/Z], [104085/Z/14/Z] Chinese Ministry of Science and Technology (2011BAI09B01), Chinese National Natural Science Foundation (81390541). The British Heart Foundation, UK Medical Research Council and Cancer Research UK provide core funding to the Clinical Trial Service Unit and Epidemiological Studies Unit at Oxford University for the project.

## Conflicts of interest

We declare that we have no conflict of interest.

## Figures and Tables

**Table 1:** Baseline characteristics of study population by age at menarche.

	Overall	Age at menarche						
		≤ 12	13	14	15	16	17	≥ 18
Number of subjects	281,491	15,251	32,426	46,984	53,132	52,286	39,817	41,595
Mean age, years	50.5	46.0	46.4	47.3	49.5	51.4	53.3	56.0
Socio-economic and lifestyle factors								
Urban resident, %	43.2	50.8	50.3	44.2	41.8	41.5	41.7	39.0
Income > 20,000 CNY/year, %^a^	40.7	44.8	44.3	42.5	41.4	39.5	38.3	35.6
No formal school education, %	25.4	23.3	21.8	22.2	24.2	25.5	27.0	30.8
Current regular smoker, %	2.3	2.4	2.2	2.2	2.3	2.3	2.4	2.3
Weekly regular drinker, %	2.1	2.4	2.0	2.0	1.9	2.0	2.2	2.4
BMI, kg/m^2^	23.7	24.5	24.2	24.0	23.8	23.6	23.5	23.2
Overweight (25–29.9 kg/m^2^), %	28.8	33.9	32.8	30.7	29.2	28.1	26.7	25.0
Obese (≥ 30 kg/m^2^), %	4.6	7.3	6.0	5.3	4.7	4.1	3.7	3.5
Leg length, cm	71.0	70.5	70.6	70.8	70.9	71.2	71.3	71.2
Total physical activity, MET-h/day	20.9	20.7	20.8	20.7	20.7	21.0	21.0	21.1
SBP, mmHg	129.4	130.5	130.1	129.9	129.7	129.3	128.7	128.5
Had prior diabetes, %	5.5	7.1	6.4	6.2	5.8	5.5	5.0	4.8
Ever use of oral contraceptive pill, %	9.8	10.5	10.4	10.3	10.0	9.8	9.7	9.1
Reproductive factors								
Nulliparous, %	1.3	1.4	1.5	1.4	1.2	1.1	1.1	1.2
Number of live births^b^	2.2	2.1	2.2	2.2	2.2	2.2	2.2	2.2
Parity ≥ 3 children, %	31.5	28.7	29.3	30.4	31.2	32.3	32.9	33.4
Age at first birth, years^b^	23.4	23.3	23.3	23.3	23.2	23.2	23.3	23.7
Never breastfed, %^b^	2.7	3.6	3.3	3.0	2.7	2.3	2.2	2.4
Breastfeeding duration per child, months^b^	14.6	13.9	14.0	14.2	14.4	14.7	14.9	15.2
Pre-menopause at baseline, %	44.6	44.9	45.3	45.8	45.2	44.4	44.0	42.7
Age at menopause, years^c^	48.3	47.5	48.0	48.1	48.2	48.3	48.4	48.5
Reproductive years, years^c^	32.3	35.6	35.0	34.1	33.2	32.3	31.4	29.9

a CNY: Chinese Yuan, 1 CNY = 0.15 US$ at Dec. 2015.

b Among parous women only.

c Among post-menopausal women only.

**Table 2:** Adjusted hazard ratios for stroke, CHD and CVD death associated with age at menarche by birth cohort^a^.

Outcome	Birth cohort	Events/HRs	Age at menarche			
			≤ 12	13–14	15–16	≥ 17
Stroke	1920s–1930s	No. events	93	722	1426	1369
		HR (95%CI)	0.84 (0.68, 1.03)	1.00 (0.93, 1.08)	1.00 (0.95, 1.06)	1.01 (0.96, 1.07)
	1940s	No. events	116	876	1681	1885
		HR (95%CI)	0.83 (0.69, 1.00)	1.00 (0.93, 1.07)	1.02 (0.97, 1.06)	0.95 (0.90, 0.99)
	1950s	No. events	248	1120	1590	1196
		HR (95%CI)	1.18 (1.04, 1.33)	1.00 (0.94, 1.06)	1.01 (0.96, 1.06)	0.96 (0.90, 1.02)
	1960s–1970s	No. events	135	536	582	263
		HR (95%CI)	1.24 (1.05, 1.47)	1.00 (0.92, 1.09)	1.12 (1.03, 1.21)	1.21 (1.07, 1.37)
CHD	1920s–1930s	No. events	97	675	1322	1207
		HR (95%CI)	0.98 (0.80, 1.19)	1.00 (0.93, 1.08)	1.02 (0.97, 1.08)	1.01 (0.95, 1.07)
	1940s	No. events	117	878	1541	1835
		HR (95%CI)	0.86 (0.72, 1.03)	1.00 (0.93, 1.07)	0.91 (0.87, 0.96)	0.91 (0.87, 0.96)
	1950s	No. events	211	1164	1579	1270
		HR (95%CI)	0.99 (0.86, 1.13)	1.00 (0.94, 1.06)	0.96 (0.91, 1.01)	0.98 (0.93, 1.04)
	1960s–1970s	No. events	142	737	753	326
		HR (95%CI)	0.97 (0.82, 1.15)	1.00 (0.93, 1.08)	1.03 (0.96, 1.11)	1.08 (0.96, 1.20)
CVD death	1920s–1940s	No. events	67	432	950	914
		HR (95%CI)	1.02 (0.80, 1.29)	1.00 (0.91, 1.10)	1.10 (1.03, 1.17)	0.99 (0.93, 1.06)
	1950s–1970s	No. events	33	169	261	202
		HR (95%CI)	0.98 (0.70, 1.38)	1.00 (0.86, 1.17)	1.10 (0.97, 1.23)	1.10 (0.95, 1.27)

a Cox model stratified by age and region and adjustment for education level, measured blood pressure, household income, smoking, alcohol drinking, BMI, physical activities (MET), prior-diabetes status and leg length.

## Appendix A Supplementary data

Supplementary material Image 1
