# Supplementary material for: Age at menarche and risk of major cardiovascular diseases: Evidence of birth cohort effects from a prospective study of 300,000 Chinese women
Source: Int J Cardiol. 2017 Jan 15;227:497–502. doi: 10.1016/j.ijcard.2016.10.115 (PMC5176087; doi:10.1016/j.ijcard.2016.10.115)
Supplement: Supplementary file 1 — Supplementary material [file mmc1.docx]

**Web-Figure: Adjusted hazard ratios for ischaemic stroke, haemorrhagic stroke associated with age at menarche, adjustment for age, region and other lifestyle risk factors.**

**Web-Table 1. Adjusted Hazards Ratios (95% CIs) for stroke, CHD and CVD deaths by different levels of adjustment**

|  | **Age at menarche** | | | | | | |
| --- | --- | --- | --- | --- | --- | --- | --- |
|  | **≤12** | **13** | **14** | **15** | **16** | **17** | **≥18** |
| **Stroke** |  |  |  |  |  |  |  |
| **Model** **1 ^*^** | **1.08 (1.00, 1.18)** | **1.00 (0.95, 1.06)** | **1.03 (0.99, 1.08)** | **1.03 (0.99, 1.07)** | **1.00 (0.96, 1.03)** | **0.95 (0.92, 1.00)** | **0.92 (0.89, 0.96)** |
| **Model 2 ^#^** | **1.07 (0.99, 1.16)** | **1.00 (0.95, 1.06)** | **1.04 (1.00, 1.09)** | **1.06 (1.02, 1.10)** | **1.04 (1.00, 1.08)** | **1.03 (0.98, 1.07)** | **1.01 (0.97, 1.05)** |
| **Model 3 ^†^** | **1.08 (0.99, 1.17)** | **1.00 (0.95, 1.06)** | **1.04 (1.00, 1.09)** | **1.06 (1.02, 1.10)** | **1.04 (1.00, 1.08)** | **1.03 (0.99, 1.07)** | **1.02 (0.98, 1.06)** |
| **Model 4 ^‡^** | **1.18 (1.04, 1.34)** | **1.00 (0.92, 1.09)** | **1.10 (1.03, 1.18)** | **1.12 (1.06, 1.18)** | **1.11 (1.05, 1.17)** | **1.08 (1.02, 1.15)** | **1.06 (1.00, 1.12)** |
| **CHD** |  |  |  |  |  |  |  |
| **Model** **1 ^*^** | **1.02 (0.94, 1.11)** | **1.00 (0.95, 1.06)** | **1.08 (1.04, 1.13)** | **1.04 (1.00, 1.08)** | **0.97 (0.93, 1.00)** | **1.02 (0.98, 1.07)** | **0.93 (0.89, 0.97)** |
| **Model 2 ^#^** | **1.01 (0.93, 1.10)** | **1.00 (0.95, 1.06)** | **1.09 (1.05, 1.14)** | **1.06 (1.02, 1.10)** | **1.00 (0.96, 1.04)** | **1.07 (1.03, 1.12)** | **0.99 (0.95, 1.03)** |
| **Model 3 ^†^** | **1.01 (0.93, 1.10)** | **1.00 (0.95, 1.06)** | **1.10 (1.05, 1.15)** | **1.05 (1.01, 1.09)** | **0.99 (0.96, 1.03)** | **1.08 (1.03, 1.12)** | **1.01 (0.97, 1.06)** |
| **Model 4 ^‡^** | **1.17 (1.03, 1.33)** | **1.00 (0.91, 1.09)** | **1.15 (1.08, 1.23)** | **1.19 (1.12, 1.26)** | **1.12 (1.06, 1.18)** | **1.15 (1.09, 1.23)** | **1.12 (1.06, 1.19)** |
| **CVD Death** |  |  |  |  |  |  |  |
| **Model** **1 ^*^** | **1.08 (0.89, 1.32)** | **1.00 (0.87, 1.15)** | **1.09 (0.98, 1.20)** | **1.18 (1.09, 1.28)** | **1.07 (0.99, 1.16)** | **1.01 (0.93, 1.11)** | **0.98 (0.90, 1.06)** |
| **Model 2 ^#^** | **1.07 (0.88, 1.31)** | **1.00 (0.87, 1.15)** | **1.09 (0.98, 1.20)** | **1.20 (1.11, 1.30)** | **1.12 (1.04, 1.21)** | **1.09 (0.99, 1.18)** | **1.05 (0.97, 1.14)** |
| **Model 3 ^†^** | **1.06 (0.87, 1.29)** | **1.00 (0.87, 1.15)** | **1.10 (1.00, 1.22)** | **1.21 (1.11, 1.31)** | **1.13 (1.05, 1.23)** | **1.12 (1.02, 1.22)** | **1.10 (1.01, 1.20)** |
| **Model 4 ^‡^** | **1.37 (1.03, 1.81)** | **1.00 (0.81, 1.23)** | **1.22 (1.05, 1.41)** | **1.35 (1.21, 1.51)** | **1.29 (1.16, 1.44)** | **1.26 (1.11, 1.42)** | **1.24 (1.11, 1.39)** |

* Model 1: Cox model stratified by age and region and adjustment for education level.

# Model 2: Model 1 + further adj. other CVD risk factors, i.e. measured blood pressure, household income, smoking, alcohol drinking, BMI, physical activities (MET), prior-diabetes status and leg length

† Model 3: Model 1 + further adj. other repro factors, i.e. menopause status, parity, age at first birth, breastfeeding duration, OC use

‡ Model 4: Only among women who never smoked or alcohol consumed, who had no diabetes or never used OC pill, by using Cox model that stratified by age and region and adjustment for education level, measured blood pressure, household income, BMI, physical activities (MET) and leg length

**Web-Table 2. Adjusted hazards Ratios (95% CIs) for stroke, CHD and CVD deaths by subgroup of area, education, BMI and menopausal status, adjustment for age, region and other lifestyle risk factors**

|  | **Age at menarche** | | | | | | |
| --- | --- | --- | --- | --- | --- | --- | --- |
|  | **≤12** | **13** | **14** | **15** | **16** | **17** | **≥18** |
| **Stroke** | 592 | 1277 | 1977 | 2577 | 2702 | 2190 | 2523 |
| Region |  |  |  |  |  |  |  |
| Rural | 1.05 (0.93, 1.18) | 1.00 (0.92, 1.09) | 1.04 (0.97, 1.10) | 1.07 (1.02, 1.13) | 1.06 (1.01, 1.11) | 1.02 (0.96, 1.07) | 1.01 (0.96, 1.07) |
| Urban | 1.09 (0.97, 1.22) | 1.00 (0.93, 1.08) | 1.04 (0.98, 1.11) | 1.05 (0.99, 1.11) | 1.03 (0.97, 1.09) | 1.05 (0.98, 1.12) | 1.01 (0.94, 1.08) |
| BMI |  |  |  |  |  |  |  |
| < 25 kg/m^2^ | 1.06 (0.94, 1.19) | 1.00 (0.92, 1.08) | 1.07 (1.01, 1.13) | 1.07 (1.01, 1.12) | 1.07 (1.02, 1.12) | 1.04 (0.98, 1.09) | 1.01 (0.96, 1.06) |
| ≥ 25 kg/m^2^ | 1.08 (0.97, 1.21) | 1.00 (0.92, 1.08) | 1.02 (0.95, 1.09) | 1.06 (1.00, 1.13) | 1.02 (0.96, 1.08) | 1.02 (0.95, 1.09) | 1.01 (0.95, 1.08) |
| Education |  |  |  |  |  |  |  |
| No | 0.88 (0.73, 1.05) | 1.00 (0.89, 1.12) | 1.00 (0.91, 1.09) | 0.97 (0.90, 1.04) | 1.02 (0.96, 1.09) | 0.97 (0.90, 1.04) | 0.95 (0.89, 1.01) |
| Yes | 1.13 (1.03, 1.24) | 1.00 (0.94, 1.07) | 1.05 (1.00, 1.11) | 1.10 (1.05, 1.15) | 1.04 (0.99, 1.09) | 1.04 (0.99, 1.10) | 1.03 (0.98, 1.09) |
| Post-menopause |  |  |  |  |  |  |  |
| No | 1.09 (0.94, 1.28) | 1.00 (0.89, 1.12) | 0.96 (0.87, 1.06) | 1.09 (0.99, 1.19) | 1.02 (0.93, 1.13) | 1.10 (0.98, 1.25) | 1.11 (0.96, 1.27) |
| Yes | 1.05 (0.96, 1.16) | 1.00 (0.94, 1.07) | 1.07 (1.02, 1.12) | 1.06 (1.02, 1.11) | 1.05 (1.01, 1.09) | 1.02 (0.98, 1.07) | 1.00 (0.96, 1.04) |
| **CHD** | 567 | 1315 | 2139 | 2579 | 2616 | 2245 | 2393 |
| Region |  |  |  |  |  |  |  |
| Rural | 1.05 (0.93, 1.19) | 1.00 (0.92, 1.09) | 1.18 (1.11, 1.25) | 1.13 (1.08, 1.20) | 1.12 (1.07, 1.18) | 1.14 (1.08, 1.21) | 1.10 (1.04, 1.16) |
| Urban | 0.98 (0.88, 1.10) | 1.00 (0.93, 1.07) | 1.04 (0.98, 1.11) | 1.01 (0.96, 1.07) | 0.89 (0.84, 0.95) | 1.03 (0.97, 1.10) | 0.90 (0.84, 0.97) |
| BMI |  |  |  |  |  |  |  |
| < 25 kg/m^2^ | 1.04 (0.92, 1.17) | 1.00 (0.93, 1.08) | 1.15 (1.08, 1.22) | 1.10 (1.05, 1.16) | 1.05 (0.99, 1.10) | 1.12 (1.06, 1.18) | 1.02 (0.96, 1.07) |
| ≥ 25 kg/m^2^ | 0.98 (0.87, 1.10) | 1.00 (0.93, 1.08) | 1.05 (0.98, 1.12) | 1.02 (0.96, 1.08) | 0.95 (0.89, 1.01) | 1.02 (0.96, 1.09) | 0.97 (0.90, 1.03) |
| Education |  |  |  |  |  |  |  |
| No | 1.14 (0.95, 1.36) | 1.00 (0.88, 1.14) | 1.28 (1.17, 1.39) | 1.21 (1.13, 1.30) | 1.23 (1.15, 1.31) | 1.26 (1.18, 1.35) | 1.14 (1.07, 1.22) |
| Yes | 0.98 (0.89, 1.07) | 1.00 (0.94, 1.06) | 1.05 (1.00, 1.10) | 1.03 (0.98, 1.07) | 0.93 (0.89, 0.97) | 1.02 (0.97, 1.08) | 0.96 (0.91, 1.01) |
| Post-menopause |  |  |  |  |  |  |  |
| No | 1.04 (0.89, 1.21) | 1.00 (0.90, 1.11) | 1.13 (1.04, 1.22) | 1.12 (1.03, 1.22) | 1.07 (0.98, 1.17) | 1.19 (1.07, 1.33) | 1.10 (0.97, 1.26) |
| Yes | 0.99 (0.90, 1.09) | 1.00 (0.94, 1.07) | 1.09 (1.03, 1.14) | 1.04 (1.00, 1.09) | 0.98 (0.94, 1.02) | 1.04 (1.00, 1.09) | 0.97 (0.93, 1.01) |
| **CVD Death** | 100 | 210 | 391 | 591 | 620 | 503 | 613 |
| Region |  |  |  |  |  |  |  |
| Rural | 1.08 (0.86, 1.37) | 1.00 (0.85, 1.18) | 1.10 (0.98, 1.24) | 1.23 (1.12, 1.35) | 1.22 (1.12, 1.33) | 1.15 (1.04, 1.26) | 1.10 (1.01, 1.21) |
| Urban | 1.05 (0.72, 1.53) | 1.00 (0.78, 1.28) | 1.07 (0.88, 1.31) | 1.15 (0.97, 1.36) | 0.82 (0.68, 0.99) | 0.93 (0.76, 1.13) | 0.93 (0.76, 1.12) |
| BMI |  |  |  |  |  |  |  |
| < 25 kg/m^2^ | 1.00 (0.76, 1.32) | 1.00 (0.83, 1.20) | 1.01 (0.88, 1.15) | 1.20 (1.08, 1.33) | 1.13 (1.02, 1.24) | 1.06 (0.95, 1.18) | 1.06 (0.96, 1.16) |
| ≥ 25 kg/m^2^ | 1.16 (0.87, 1.54) | 1.00 (0.81, 1.23) | 1.23 (1.06, 1.43) | 1.20 (1.05, 1.38) | 1.13 (0.99, 1.30) | 1.16 (1.00, 1.35) | 1.01 (0.87, 1.18) |
| Education |  |  |  |  |  |  |  |
| No | 0.93 (0.68, 1.28) | 1.00 (0.81, 1.23) | 1.17 (1.02, 1.35) | 1.25 (1.12, 1.40) | 1.17 (1.05, 1.30) | 1.12 (1.00, 1.27) | 1.10 (0.99, 1.23) |
| Yes | 1.18 (0.92, 1.52) | 1.00 (0.83, 1.20) | 1.03 (0.90, 1.19) | 1.18 (1.04, 1.33) | 1.10 (0.98, 1.24) | 1.08 (0.95, 1.23) | 1.05 (0.93, 1.19) |
| Post-menopause |  |  |  |  |  |  |  |
| No | 1.31 (0.81, 2.12) | 1.00 (0.67, 1.50) | 1.11 (0.82, 1.50) | 1.38 (1.05, 1.82) | 1.13 (0.82, 1.56) | 1.30 (0.90, 1.88) | 1.45 (0.99, 2.13) |
| Yes | 1.02 (0.82, 1.27) | 1.00 (0.87, 1.16) | 1.08 (0.97, 1.20) | 1.19 (1.09, 1.29) | 1.12 (1.03, 1.21) | 1.07 (0.98, 1.17) | 1.03 (0.95, 1.12) |

**Web-Table 3. Baseline characteristics of study population by year of birth**

| \| **Risk factor** \| **Birth year** \| **Overall** \| **Age at menarche** \| \| \| \| \| \| \| \| --- \| --- \| --- \| --- \| --- \| --- \| --- \| --- \| --- \| --- \| \| **≤12** \| **13** \| **14** \| **15** \| **16** \| **17** \| **≥18** \| \| **Household income >20,000 CNY/year, %^†^** \| **1920s-1930s** \| 28.1 \| 33.1 \| 35.3 \| 30.3 \| 28.9 \| 28.5 \| 26.7 \| 27.0 \| \| **1940s** \| 37.6 \| 42.6 \| 41.8 \| 38.8 \| 39.1 \| 37.8 \| 37.3 \| 34.3 \| \| **1950s** \| 42.8 \| 47.4 \| 46.4 \| 45.5 \| 45.1 \| 43.5 \| 42.6 \| 40.2 \| \| **1960s-1970s** \| 43.4 \| 46.1 \| 45.1 \| 44.0 \| 42.2 \| 39.8 \| 38.4 \| 35.2 \| \| **No formal education, %** \| **1920s-1930s** \| 58.2 \| 52.7 \| 52.9 \| 53.9 \| 57.8 \| 59.8 \| 63.1 \| 66.7 \| \|  \| **1940s** \| 31.2 \| 30.4 \| 26.6 \| 28.6 \| 31.4 \| 32.8 \| 34.1 \| 39.4 \| \|  \| **1950s** \| 29.2 \| 27.8 \| 26.7 \| 26.6 \| 28.5 \| 30.3 \| 31.6 \| 34.9 \| \|  \| **1960s-1970s** \| 8.7 \| 9.0 \| 7.6 \| 7.6 \| 8.5 \| 9.4 \| 10.0 \| 12.7 \| \| **Current regular smoker, %** \| **1920s-1930s** \| 6.8 \| 6.4 \| 5.6 \| 6.4 \| 6.5 \| 6.8 \| 6.5 \| 6.6 \| \|  \| **1940s** \| 3.3 \| 4.5 \| 3.4 \| 3.9 \| 3.8 \| 3.8 \| 3.6 \| 3.7 \| \|  \| **1950s** \| 1.9 \| 2.1 \| 2.1 \| 1.8 \| 1.9 \| 1.9 \| 2.0 \| 2.0 \| \|  \| **1960s-1970s** \| 0.9 \| 0.8 \| 0.8 \| 0.8 \| 0.9 \| 0.9 \| 1.0 \| 0.8 \| \| **Weekly regular drinker, %** \| **1920s-1930s** \| 3.0 \| 3.4 \| 2.6 \| 2.4 \| 2.1 \| 3.0 \| 3.2 \| 3.3 \| \|  \| **1940s** \| 2.4 \| 2.4 \| 2.6 \| 2.6 \| 2.4 \| 2.1 \| 2.4 \| 2.4 \| \|  \| **1950s** \| 2.2 \| 2.6 \| 2.2 \| 2.0 \| 1.9 \| 2.2 \| 2.2 \| 2.5 \| \|  \| **1960s-1970s** \| 1.7 \| 1.9 \| 1.6 \| 1.6 \| 1.6 \| 1.6 \| 1.8 \| 2.0 \| \| **Overweight/Obese (BMI ≥25 kg/m^2^), %** \| **1920s-1930s** \| 32.5 \| 39.6 \| 40.5 \| 35.4 \| 34.1 \| 33.0 \| 29.4 \| 27.9 \| \| **1940s** \| 37.7 \| 44.7 \| 45.9 \| 41.1 \| 39.6 \| 38.5 \| 35.4 \| 32.2 \| \| **1950s** \| 37.3 \| 44.8 \| 43.2 \| 41.2 \| 39.0 \| 36.5 \| 34.6 \| 32.2 \| \| **1960s-1970s** \| 29.0 \| 37.1 \| 31.7 \| 29.4 \| 26.7 \| 25.2 \| 24.0 \| 23.1 \| \| **Total physical activity, MET-h/day** \| **1920s-1930s** \| 12.4 \| 11.2 \| 11.4 \| 11.3 \| 11.7 \| 11.6 \| 11.8 \| 12.0 \| \| **1940s** \| 17.0 \| 16.1 \| 16.3 \| 16.3 \| 16.3 \| 16.6 \| 16.9 \| 17.3 \| \| **1950s** \| 21.4 \| 20.5 \| 20.5 \| 20.4 \| 20.6 \| 20.9 \| 20.8 \| 20.8 \| \| **1960s-1970s** \| 25.3 \| 25.1 \| 25.3 \| 25.2 \| 25.1 \| 25.5 \| 25.7 \| 25.9 \| \| **SBP, mmHg** \| **1920s-1930s** \| 144.2 \| 144.8 \| 145.8 \| 144.5 \| 145.3 \| 144.3 \| 144.0 \| 144.0 \| \|  \| **1940s** \| 137.2 \| 140.3 \| 139.5 \| 139.5 \| 138.7 \| 138.4 \| 137.5 \| 136.9 \| \|  \| **1950s** \| 129.6 \| 132.2 \| 131.2 \| 131.3 \| 131.1 \| 130.2 \| 129.3 \| 128.8 \| \|  \| **1960s-1970s** \| 121.0 \| 121.5 \| 121.3 \| 120.7 \| 120.5 \| 120.1 \| 119.5 \| 119.5 \| \| **Had prior diabetes, %** \| **1920s-1930s** \| 11.8 \| 13.5 \| 14.7 \| 14.1 \| 11.9 \| 12.2 \| 10.8 \| 10.1 \| \|  \| **1940s** \| 9.3 \| 11.6 \| 12.5 \| 11.4 \| 10.5 \| 10 \| 8.7 \| 7.9 \| \|  \| **1950s** \| 5.1 \| 7.6 \| 6.5 \| 6.3 \| 5.9 \| 5.4 \| 4.8 \| 4.6 \| \|  \| **1960s-1970s** \| 2.1 \| 3.0 \| 1.9 \| 2.0 \| 1.9 \| 1.6 \| 1.7 \| 1.8 \| \| **Parity ≥ 3children, %** \| **1920s-1930s** \| 86.6 \| 83.1 \| 84.5 \| 86.6 \| 86.6 \| 88.0 \| 88.5 \| 86.0 \| \|  \| **1940s** \| 57.3 \| 61.0 \| 59.9 \| 62.4 \| 63.1 \| 63.8 \| 63.4 \| 63.7 \| \|  \| **1950s** \| 21.8 \| 20.5 \| 22 \| 22.3 \| 22.7 \| 23.2 \| 23.3 \| 24.0 \| \|  \| **1960s-1970s** \| 9.9 \| 8.7 \| 8.9 \| 9.1 \| 9.7 \| 10.2 \| 10.7 \| 11.0 \| \| **Never breastfed, %*** \| **1920s-1930s** \| 1.4 \| 2.9 \| 1.7 \| 2.3 \| 1.5 \| 1.3 \| 1.3 \| 1.4 \| \|  \| **1940s** \| 1.4 \| 1.9 \| 2.2 \| 1.4 \| 1.5 \| 1.4 \| 1.0 \| 1.2 \| \|  \| **1950s** \| 2.8 \| 3.6 \| 3.6 \| 3.2 \| 2.7 \| 2.3 \| 2.3 \| 2.1 \| \|  \| **1960s-1970s** \| 3.7 \| 4.7 \| 4.1 \| 3.6 \| 3.6 \| 3.1 \| 2.9 \| 3.6 \|   *† CNY: Chinese Yuan, 1 CNY= 0.15 US$ at Dec. 2015; * Among parous women only* |  |  |  |  |  |  |  |  |
| --- | --- | --- | --- | --- | --- | --- | --- | --- | --- | --- | --- | --- | --- | --- | --- | --- | --- | --- | --- | --- | --- | --- | --- | --- | --- | --- | --- | --- | --- | --- | --- | --- | --- | --- | --- | --- | --- | --- | --- | --- | --- | --- | --- | --- | --- | --- | --- | --- | --- | --- | --- | --- | --- | --- | --- | --- | --- | --- | --- | --- | --- | --- | --- | --- | --- | --- | --- | --- | --- | --- | --- | --- | --- | --- | --- | --- | --- | --- | --- | --- | --- | --- | --- | --- | --- | --- | --- | --- | --- | --- | --- | --- | --- | --- | --- | --- | --- | --- | --- | --- | --- | --- | --- | --- | --- | --- | --- | --- | --- | --- | --- | --- | --- | --- | --- | --- | --- | --- | --- | --- | --- | --- | --- | --- | --- | --- | --- | --- | --- | --- | --- | --- | --- | --- | --- | --- | --- | --- | --- | --- | --- | --- | --- | --- | --- | --- | --- | --- | --- | --- | --- | --- | --- | --- | --- | --- | --- | --- | --- | --- | --- | --- | --- | --- | --- | --- | --- | --- | --- | --- | --- | --- | --- | --- | --- | --- | --- | --- | --- | --- | --- | --- | --- | --- | --- | --- | --- | --- | --- | --- | --- | --- | --- | --- | --- | --- | --- | --- | --- | --- | --- | --- | --- | --- | --- | --- | --- | --- | --- | --- | --- | --- | --- | --- | --- | --- | --- | --- | --- | --- | --- | --- | --- | --- | --- | --- | --- | --- | --- | --- | --- | --- | --- | --- | --- | --- | --- | --- | --- | --- | --- | --- | --- | --- | --- | --- | --- | --- | --- | --- | --- | --- | --- | --- | --- | --- | --- | --- | --- | --- | --- | --- | --- | --- | --- | --- | --- | --- | --- | --- | --- | --- | --- | --- | --- | --- | --- | --- | --- | --- | --- | --- | --- | --- | --- | --- | --- | --- | --- | --- | --- | --- | --- | --- | --- | --- | --- | --- | --- | --- | --- | --- | --- | --- | --- | --- | --- | --- | --- | --- | --- | --- | --- | --- | --- | --- | --- | --- | --- | --- | --- | --- | --- | --- | --- | --- | --- | --- | --- | --- | --- | --- | --- | --- | --- | --- | --- | --- | --- | --- | --- | --- | --- | --- | --- | --- | --- | --- | --- | --- | --- | --- | --- | --- | --- | --- | --- | --- | --- | --- | --- | --- | --- | --- | --- | --- | --- | --- | --- | --- | --- | --- | --- | --- | --- | --- | --- | --- | --- | --- | --- | --- | --- | --- | --- | --- | --- | --- | --- | --- | --- | --- | --- | --- | --- | --- | --- | --- | --- | --- | --- | --- | --- | --- | --- | --- | --- | --- | --- | --- | --- | --- | --- | --- | --- | --- |
